# Phylogenic inference using alignment-free methods for applications in microbial community surveys using 16s rRNA gene

**DOI:** 10.1371/journal.pone.0187940

**Published:** 2017-11-14

**Authors:** Yifei Zhang, Alexander V. Alekseyenko

**Affiliations:** 1 Department of Medicine, New York University School of Medicine, New York, NY, United States of America; 2 Biomedical Informatics Center, Departments of Public Health Sciences and Oral Health Sciences, Program for Human Microbiome Research, Medical University of South Carolina, Charleston, SC, United States of America; Wilfrid Laurier University, CANADA

## Abstract

The diversity of microbiota is best explored by understanding the phylogenetic structure of the microbial communities. Traditionally, sequence alignment has been used for phylogenetic inference. However, alignment-based approaches come with significant challenges and limitations when massive amounts of data are analyzed. In the recent decade, alignment-free approaches have enabled genome-scale phylogenetic inference. Here we evaluate three alignment-free methods: ACS, CVTree, and Kr for phylogenetic inference with 16s rRNA gene data. We use a taxonomic gold standard to compare the accuracy of alignment-free phylogenetic inference with that of common microbiome-wide phylogenetic inference pipelines based on PyNAST and MUSCLE alignments with FastTree and RAxML. We re-simulate fecal communities from Human Microbiome Project data to evaluate the performance of the methods on datasets with properties of real data. Our comparisons show that alignment-free methods are not inferior to alignment-based methods in giving accurate and robust phylogenic trees. Moreover, consensus ensembles of alignment-free phylogenies are superior to those built from alignment-based methods in their ability to highlight community differences in low power settings. In addition, the overall running times of alignment-based and alignment-free phylogenetic inference are comparable. Taken together our empirical results suggest that alignment-free methods provide a viable approach for microbiome-wide phylogenetic inference.

## Introduction

Historically, bacterial systematics has been a difficult problem because bacteria lack morphological features, which would be easy to characterize. However, after Carl Woese and collaborators started creating phylogenies based on small subunit (SSU) ribosomal RNA (rRNA) sequences[1], sequence-based phylogenies have been accepted as the standard in creating the Tree of Life inference by many biologists. Morphology-based taxonomies have been almost entirely superseded by sequencing-based systematics approaches.

The overwhelming explosion in the amount of genetic sequence data available for research has been brought about in the last decade by advances in molecular sequencing technologies. Traditional analysis pipelines for such data involve alignment-based methods such as BLAST [2], PyNAST [3], NAST [4], and SINA [5]. The ability to perform high-throughput sequencing of marker genes, such as 16S rRNA gene, has enabled en masse microbial community surveys. The studies in this area have yielded valuable information for characterization of the human microbiome and for understanding its role in many diseases such as irritable bowel syndrome, chronic obstructive pulmonary disease [6], obesity [7, 8], diabetes [9], psoriasis [10], cancer [11], and depression [12]. From the sequencing-informatics perspective, sequence alignment has remained an important algorithmic approach in microbiomics. The challenge of alignment-based approaches is in dealing with the volume of the data in terms of computation time and data management. Many analyses have reduced the severity of the problem by clustering individual sequences into operational taxonomical units (OTUs). Doing so significantly reduces the number of sequences that need to be aligned and have their phylogenetic relationships inferred, typically from millions of raw sequences to a few thousand representative sequences for the OTUs. However, many concerns still exist about the ability to infer reliable pairwise alignments, and subsequently to infer multiple sequence alignments necessary for phylogenetic inference.

In whole-genome phylogenetic inference, alignment-free approaches have been proposed over the past three decades [13, 14], bringing with it an explosion of reports on new alignment-free approaches in the last 10 years [15]. These approaches typically forego the necessity to infer multiple sequence alignments for phylogenetic inference by considering evolutionary models built on more complex characters than single nucleotides or amino acids. A majority of these models deal with quantitation of specific *k*-mer words, which are treated as unitary traits. The idea of k-mer, or perfectly matched strings of selected length, have been used by groups such as Blasdell and Gibbs et al in tree inferences for proteins and nucleotides [16]. By ascertaining the frequency or presence and absence of these k-mers, a trait table or an evolutionary distance matrix can be created to facilitate phylogenetic inference.

In this study, we aim to extend the application of three alignment-free methods, ACS [17], CVTree [18], and Kr [19], to phylogenetic inference with 16S rRNA gene data. We evaluate the performance of these methods in terms of accuracy and computation time. For reference, we compare these methods to phylogenies derived from traditional alignment-based methods. Namely, we infer phylogenies by FastTree [20] and RAxML [21] built on PyNAST [3] or MUSCLE [22] alignments, which are approaches widely used for 16S rRNA gene analysis in the microbiome community (for example, see [23], [24], [25]). We use Greengenes taxonomy as a gold standard to evaluate correctness of the trees inferred by all methods. We also utilize stool-derived data from the Human Microbiome Project Data Analysis and Coordination Center (hmpdacc.org) to re-simulate the data and test the performance of these methods for gut community studies, which are the most widely funded and studied to date.

## Methods

### Data

To re-simulate realistic datasets for method evaluation purposes, we have obtained sequences from the Greengenes 16S rRNA gene database [26] and from the Human Microbiome Project (HMP) Data Analysis and Coordination Center (DACC) [27]. We re-simulate human stool communities by sampling OTUs from HMP datasets. We mimicked the typical characteristics of a microbiome datasets in terms of the number and the representation of OTUs by resampling from existing stool microbiota. Each re-simulated community consisted of approximately 5,000 OTUs drawn randomly from OTUs present in HMP stool samples. This was done by filtering Greengenes to only obtain species occurring in the stool subsets. The number of OTUs was chosen as typical for evaluation of microbiome datasets.

### Alignment-free phylogenetic inference methods

Next we describe the alignment-free methods for generating pairwise distance matrices.

Average common substring approach (ACS) [17] is based on matching statistics, and computes locally maximal common substrings between two sequences, *X* and *Y*. The longest common subsequence for each position *i* in a sequence *X* is defined as the longest identical string in sequence *Y* starting with the letter *X[i]*. The average length of these longest common substrings is calculated as a reflection of similarity between *X* and *Y*. For example, given two sequences *X* = ‘TCTGA’ and *Y* = ‘CCTGT’, the length would be 3. ACS is normalized to account for the sequence lengths, resulting in a similarity measure between *X* and *Y*. To convert it to a distance, the inverse is taken and a correction term is subtracted to ensure that the distance between identical sequences is 0. The running time of ACS distance computation algorithm is linear in the length of the longest sequence. The efficiency of the algorithm allows pairwise distance computation for a moderate to large number of sequences.

CVTree [18] uses a composition vector approach. Given a DNA or amino acid sequence of *l*, it finds the frequency of appearance of overlapping strings of a fixed length k in the sequence. The frequency can also be divided by the total number (*l*-k+1) of k-strings to obtain the probability of that string appearing in the protein. The collection of frequencies and probabilities can be thought of as the result of mutations and selection forces with each k-string as a unit. While mutations happen randomly at the molecular level, selections are the forces that drive evolution. Neutral mutations account for some randomness found in the k-string composition. They are referred to as the random background and must be subtracted from the simple counting results to find the selective changes. After counting for all strings of k-1 mers and k-2 mers, the probability of appearance of k-string can be predicted using a Markov assumption. The difference between the actual counts and predicted counts is then used as the component of a new “normalized” CV, and pairwise distances between the new CVs are computed to generate a dissimilarity matrix.

Kr method [19] attempts to estimate pairwise sequence similarity based on the lengths of exact matches between pairs of sequences at each position. Kr therefore uses a quantity similar in concept to ACS, but computes the average shortest unique substring while ACS uses the average common string. For example, given two sequences *X* = ‘TCTGA’ and *Y* = ‘TCGGT’, at every position *i* along the sequence *X*, we find the shortest substring starting at *i* that is not found in *Y*. In this case, ‘TCT’ is the shortest string starting at position 1 that is absent from *Y*. The average length of these absent substrings is calculated to infer similarity between the two sequences. Because this method is based on a mathematical model of DNA sequence evolution, it is restricted to DNA sequences. However, this method is reported to be more accurate than model-free approaches [19].

### Evaluation methodology

**Fig 1:**
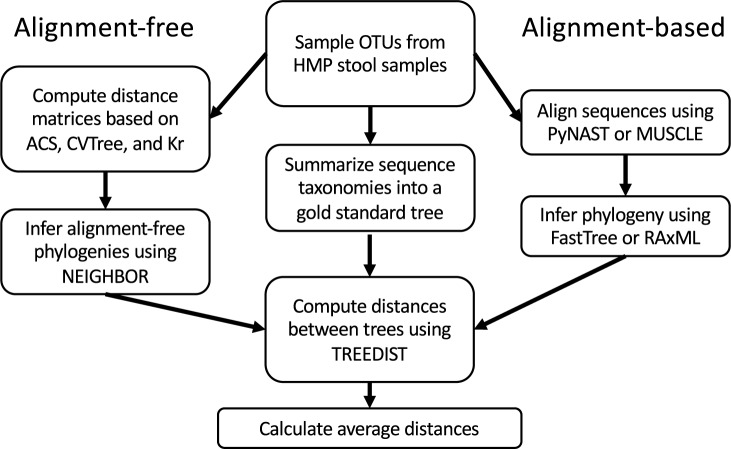
Method evaluation flowchart. Subsets of 5000 representative sequences for operational taxonomical units (OTU) of stool samples from the Human Microbiome Project have been drawn. Trees are obtained in three ways: alignment-free method (left), golden standard based on taxomic identity (middle), and alignment-based method using traditional methods (right). The trees are then compared to one another using Treedist, with the distance representing how similar they are to each other (lower number denotes greater similarity). Ten replicates of each comparison have been performed and the results are averaged.

A general outline of the methods used in the comparisons is provided in **Fig 1**. We evaluate the alignment-free methods relative to the common alignment-based inference approaches on re-simulated stool communities.

After we filtered the OTUs from the HMP dataset, the taxonomic identity of these OTUs have been used to generate the gold standard, with their respective reference sequences used for tree inference. The obtained trees are then compared using PHYLIP program TREEDIST. The parameters used for running individual programs can be found in **S1 Text**.

To compare the alignment-free methods to the state-of-the-art methods in microbiome community, we have included trees obtained from alignment-based methods. Specifically, for alignment method, we used PyNAST [3] and MUSCLE [22]. PyNAST is a widely used alignment-based tool for 16S rRNA gene analysis. It takes a set of sequence and aligns it against a template alignment, and gives the output of a multiple sequence alignment with the same number of positions as the template alignment. MUSCLE, short for Multiple Sequence Comparison by Log-Expectation, was shown to achieve similar accuracy and improved speed compared to the other major methods for alignment of nucleotide and protein sequences. After obtaining the alignment from either PyNAST or MUSCLE, we then use maximum likelihood (ML) methods FastTree [20] and RAxML [21] for inferring the phylogeny. FastTree uses the alignment to produce a maximum-likelihood phylogenetic tree by iterative rearrangement of branches. It uses the Jukes-Cantor or generalized time-reversible (GTR) models of nucleotide evolution. RAxML is the current leading method for large-scale ML estimation, and it is shown to yield the best ML scores compare to many other methods. These alignment-based trees have been included in the tree distance computation with alignment-free trees.

In the second comparison, we created consensus trees of each group to compare the accuracy of alignment-free and alignment-based trees as a whole. We used the same re-simulation strategy as in the first comparison. The PHYLIP program CONSENSE is used to create consensus trees from individual trees. We first computed the consensus tree of the alignment-free trees using CONSENSE. After resolving multifurcations in the consensus tree by adding zero-length branches, we next used FITCH to estimate the branch lengths of the consensus tree under the three alignment free alternatives. The final branch lengths have been computed as the average branch length for ACS, Kr and CVTree. Similarly, we also obtained consensus trees for the alignment-based methods. We created a consensus for each alignment method (such as a consensus with MUSCLE-based alignment, namely MUSCLE + FastTree and MUSCLE + RAxML) and for each tree inference (such as a consensus with FastTree inference, namely MUSCLE + FastTree and PyNAST + FastTree). We also created a consensus tree of all alignment-based trees. The consensus trees have been included in the tree distance computation as in the other comparisons.

Taxonomic tree generator algorithm. To establish a taxonomic tree, we created a “tree generator” program that generates the tree based on the taxonomy information provided by Greengenes, which would serve as the “gold standard” in evaluating the accuracy of the trees. The program does so by first storing the taxonomy information in the form of a matrix, which includes the name of each sequence and its taxonomy information. It then goes through the taxonomy information level by level (i.e. domain, kingdom, phylum…) and compares the sequences to one another at each level. The resulting comparison results are written in the required tree structure.

All experiments presented in this manuscript were run on the Asclepius Compute Cluster at the Center for Health Informatics and Bioinformatics (CHIBI) at New York University Langone Medical Center (http://www.nyuinformatics.org).

## Results

**Fig 2:**
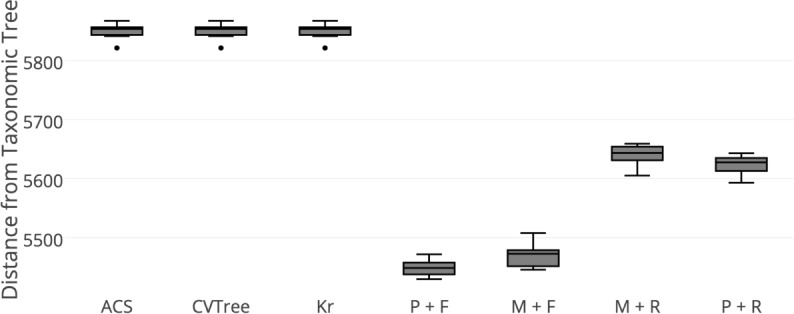
Tree distances of alignment-free methods and alignment-based methods relative to the gold standard taxonomic trees. Ten replicate subsets of sequences from Greengenes have been obtained and phylogenies inferred using alignment-free methods (ACS, CVTree, and Kr) and alignment-based methods (PyNAST or MUSCLE-based alignment, FastTree or RAxML inference). TREEDIST distances between the phylogenies inferred by each method as well as the taxonomic gold standard have been computed. Smaller distances indicate better resemblance of the taxonomy in the corresponding inferred phylogenies. Sequences from HMP derived stool samples have been used to compare all the methods. Distances across the replicates are reported. P = PyNAST, M = MUSCLE, F = FastTree, R = RAxML.

The distances to taxonomic tree for each method, across the 10 replicates, are reported in the form of a box plot in **Fig 2**. We compare the methods first within each group and than to each other.

### Alignment-free methods perform comparably in recovering the taxonomic tree

First, we evaluate the relative performance of the three alignment-free methods in recovering the taxonomic tree. On average, all three methods perform comparably with no clear winner. Interestingly, even though the overall similarity of the alignment-free trees to the taxonomic tree is comparable, there is a lot of discordance among the trees inferred by the three alignment-free methods (**S1 Fig**). This discordance suggests that the distance metrics capture different aspects of the evolutionary process.

### PyNAST and FastTree are superior among alignment-based methods

We now compare the alignment-based methods. Alignment by PyNAST with inference by FastTree clearly produces the most accurate tree. This is followed by alignment by MUSCLE with inference by FastTree. The other two RAxML-based inferences lag behind, with PyNAST/RAxML performing better than MUSCLE/RAxML.

### Alignment-free methods lag alignment-based methods in recovering the taxonomic tree

Alignment-based trees bear more resemblance of the taxonomic trees than the alignment-free trees, which perform similarly to one another. FastTree obtained from PyNAST alignment is the most accurate, followed by FastTree from MUSCLE alignment. FastTree inference thus appears to produce the closest tree to the taxonomic tree.

### Consensus tree from alignment-free methods is superior to consensus tree from alignment-based methods in recovering the taxonomic tree

**Fig 3:**
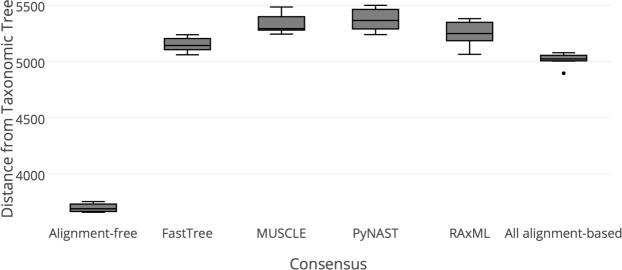
Tree distances of consensus tree of alignment-free methods, consensus of MUSCLE-based alignments, consensus of PyNAST-based alignments, consensus of FastTree inference, consensus of RAxML inference, consensus of all alignment-based methods relative to gold standard taxonomic tree. Using the same subsets as in Fig 2, a consensus tree based on the three alignment-free methods has been built. Similarly, consensus trees based on different combinations of alignment-based methods are built. TREEDIST distances across the replicates are reported; smaller distances indicate better resemblance of the consensus tree to the gold standard taxonomic tree.

Although individual trees from alignment-free method on average perform worse than trees from alignment-based method, we investigate how a consensus tree built from alignment-free methods compares to alignment-based method in terms of performance. The large discordance we observe before among alignment-free methods might now result in a more powerful consensus compared to the consensus of the more similar alignment-based methods. Therefore, we created a consensus tree from ACS, CVTree, and Kr, as well as consensus trees of a combination of PyNAST, MUSCLE, FastTree, and RAxML. We use the same sequence subsets as in the previous comparison to evaluate the similarity of a consensus trees with the taxonomic tree. The result is consistent among the replicates, and the distances are reported in **Fig 3**. In all the cases, the consensus of alignment-free results in smaller distances to the gold standard taxonomic tree, showing superior performance to all the other consensus trees, including the consensus tree of all the alignment-based methods.

### Running time

For alignment-based methods, the timing is broken down into two components, with the first getting alignment from either PyNAST or MUSCLE, and the second performing the inference using FastTree or RAxML. PyNAST alignments took approximately 6 minutes, and MUSCLE took about 3 hours. FastTree spent approximately 45–60 minutes generating the tree, while RAxML took on average 25 hours. Therefore the total time varied from one hour (PyNAST/FastTree) to up to 28 hours (MUSCLE/RAxML).

For alignment-free methods, the timing is composed of initially building distance matrices, and then building trees from the distances matrices using NEIGHBOR. Building the distance matrices with CVTree took approximately 5 minutes, while building the distance matrices for ACS and Kr took approximately 5 hours. Neighbor took on average 1 to 2 hours to build a tree for each of these methods. The total time varied from a little over 1 hour (CVTree/Neighbor) to 7 hours (ACS/Neighbor and Kr/Neighbor).

Building the consensus tree from individual trees took approximately 3 minutes regardless of the tree types.

### Alignment-free methods result in better separation of relevant experimental groups in PERMANOVA analyses

A commonly adopted approach for analysis of microbiome data is to perform permutational multivatiate analysis of variance analysis (PERMANOVA) to associate the microbial communities with experimental variables [28]. This analysis uses arbitrary distances to compute pseudo-F statistic for the factor of interest and assess its significance via permutations. The choice of a distance for PERMANOVA analysis may have an effect on its power to detect differences. Phylogenetically-based distances, such as weighted Unifrac [29] have been shown to have superior performance in such analyses. The weighted Unifrac distance takes a phylogenetic tree as input to determine the differences between communities. A choice of a phylogenetic inference method thus affects the PERMANOVA inference with Unifrac distances.

**Fig 4:**
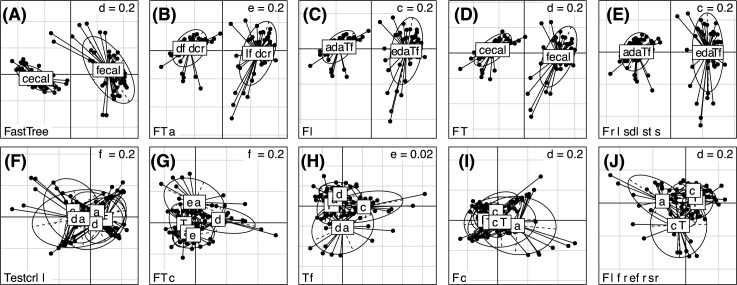
**Principal coordinates analysis of the weighted unifrac distances computed with (A) FASTTREE, (B) ACS, (C) Kr, (D) CV, (E) alignment free consensus phylogenies and grouped by sample location, and grouped by antibiotic treatment type C-control, P-penicillin, V-vancomycin, T-tetracyclin, VP-vancomycin and penicillin after centering according to sample location with (F) FastTree, (G) ACS, (H) Kr, (I) CV, and (J) alignment free consensus phylogenies**.

We re-analyze the data from a recent study of the effect of sub-therapeutic antibiotic treatment (STAT) on the microbiota [7]. In this study, mice have been continuously administered low doses of antibiotics (penicillin, vancomycin, tetracyclin, and vancomycin-penicillin cocktail) in their drinking water or acidified water. At sacrifice the fecal and cecal contents of these mice have been analyzed. We computed pairwise distances between all samples using weighted Unifrac distances with phylogenies obtained by each of the alignment-free methods considered here and the FastTree with PyNAST tree used to analyze these data originally. We have used PERMANOVA to associate the location (cecal or fecal) from which the sample has been obtained, and the location in conjunction with the treatment type. **Table 1** summarizes the estimated effect sizes and significance values for this analysis. We note that the estimated effect size based on FastTree weighted Unifrac distances is higher than for any of the alignment-free methods or their consensus. However, when the treatment effect is considered, the estimated effect is larger for the alignment-free methods, and largest for the alignment-free consensus. This results holds regardless of which effect size measurement is used—the coefficient of determination (R^2^) or omega squared (ω^2^), which has been shown to be superior for microbiome studies [30]. All of the results are statistically significant. This suggests that while FastTree phylogenies help improve separation for large effect sizes, the alignment-free phylogenies may be better for narrowing in on smaller yet important effects (**Fig 4**).

**Table 1:** Significance and effect size estimates for PERMANOVA testing (10,000 permutations) of the association of the microbiome and experimental variables.

	R^2^	ω^2^	P-value
Location			
FastTree	0.546	0.536	<0.0001
ACS	0.362	0.351	<0.0001
Kr	0.370	0.359	<0.0001
CV	0.464	0.454	<0.0001
Consensus (ACS, Kr, CV)	0.297	0.286	<0.0001
**Antibiotic type | Location**			
FastTree	0.098	0.056	<0.0001
ACS	0.127	0.084	<0.0001
Kr	0.121	0.079	<0.0001
CV	0.111	0.068	<0.0001
Consensus (ACS, Kr, CV)	0.138	0.095	<0.0001

### Empirical results confirm better discriminatory power of alignment-free consensus

**Fig 5:**
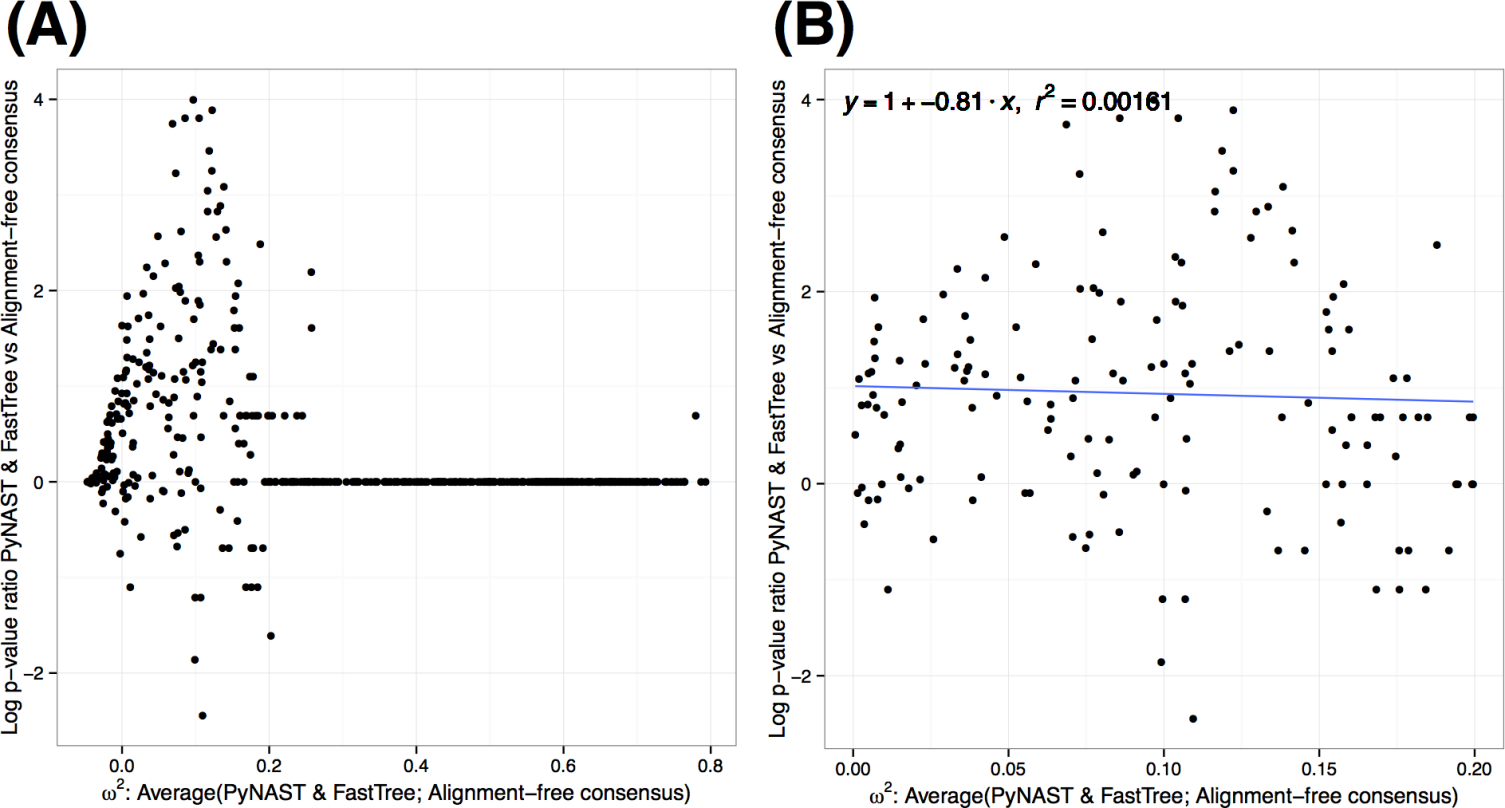
Comparison of effect size in PERMANOVA analysis with alignment-free consensus tree and with an alignment-based method. We have simulated data with various effect sizes by resampling permuted communities from control fecal specimens of the STAT dataset. ω^2^ has been computed in comparison of the weighted Unifrac distances based on both trees. The plots show the log ratio of the p-value vs. the mean of the estimated effect sizes. In (A) the entire range of effect sizes is considered and we note that at high effect sizes there is agreement in inference based on the two trees. In (B) small effect sizes are examined closely. Here the significant positive intercept of the regression indicates that alignment-free consensus phylogeny results in lower p-value than phylogeny inferred with FastTree based on PyNAST alignment.

We have further explored the question of whether alignment-free consensus tree helps to distinguish small effect sizes in PERMANOVA analysis of the weighted Unifrac distances. Here we considered the control fecal specimens from the STAT dataset and simulated additional datasets for comparison by permuting a prescribed number of OTU labels (250, 500, 1,000, 1,500 or 3,000 OTUs were perturbed). This allowed us to obtain datasets with varying effect sizes without affecting the underlying phylogenetic structure between OTUs. We have analyzed the resulting simulated datasets for differences from the original data using PERMANOVA with weighted Unifrac distances based on the alignment-free consensus and PyNAST with FastTree phylogeny (**Fig 5**). We note that when effect sizes are large (**Fig 5A**), the inference obtained with both trees are almost identical. However, in the cases with small effect sizes, we note that the p-values obtained with alignment-free phylogenies tend to be lower, indicating a higher power to detect small effects (**Fig 5B**).

## Discussion

Our comparisons show that alignment-free methods are not inferior to alignment-based methods in giving accurate results in the form of robust phylogenic trees. In fact, consensus ensembles of alignment-free phylogenies are far superior to those based on alignment-based methods in correctly recovering taxonomic relationships.

In comparing the distances of taxonomic trees and trees derived from alignment-free methods, it appears that even though the three alignment-free methods have similar accuracy, they capture different aspects of the evolutionary process, resulting in different tree topologies. Alignment-based methods, on the other hand, are much more similar to one another. Thus, it is likely that alignment-free methods approximate the evolutionary features using a variety of techniques that capture different characteristics of the true evolutionary process.

When each individual alignment-free tree is compared to an alignment-based tree from a combination of PyNAST, MUSCLE, RAxML and FastTree, the performance is lower, with the tree from PyNAST/FastTree showing the greatest accuracy. However, the consensus of the alignment-free methods performs far superiorly to the consensus from alignment-based methods in terms of accuracy. The greater dissimilarity among the alignment-free trees as noted above results in less resolved consensus trees, which are more reflective of the taxonomic gold standard.

Moreover, alignment-free methods appear to be superior at deriving better separation of small effect sizes. We are able to show through our re-analysis of a dataset on sub-therapeutic antibiotics that alignment-free phylogenies facilitated better separation of the effect of the relevant treatment. Similarly, our analysis of simulated datasets of fecal specimens with varying effect sizes also confirmed that alignment-free phylogenies are more sensitive at detecting small effects than their alignment-based counterparts.

Overall, the running times of alignment-based and alignment-free phylogenetic inference are comparable. In terms of running time, CVTree is the fastest method, which takes only approximately 5 minutes to generate the distance matrices while maintaining similar accuracy to the slower alternatives. The fact that ACS and Kr are slow in building distance matrices reflects on potential algorithmic and implementation inefficiencies, rather than inferior computational complexity. This inefficiency should be possible to overcome with additional software engineering. Creating the consensus tree from the individual trees requires a relatively short running time. While the total time for building the consensus tree (including building the individual trees) is significantly longer than for PyNAST/FastTree, the accuracy achieved may make it a worthwhile endeavor. It is also worth noting that while PyNAST and FastTree were considerably faster than the alignment-free methods, the other combinations took significantly longer times. In particular, MUSCLE took about three hours longer than PyNAST and RAxML, averaging about 24 hours (versus only an hour for FastTree). The result of PyNAST/FastTree, as noted above, was also better than any other alignment-based combination.

Although alignment-free methods are able to overcome the limitations of alignment-based methods as discussed previously, they are often passed over because of the presumed compromise in accuracy. However, our study showed that while individual tree may have decreased accuracy, their consensus trees may actually achieve greater accuracy. Most importantly as demonstrated by our re-analysis of the sub-therapeutic antibiotic treatment dataset, some studies may benefit from utilization of alignment-free trees, which may help to bring out important smaller effects.

## Supporting information

S1 Fig Average tree distances between alignment-free methods and the taxonomic gold-standard in the data from HMP stool samples. (PDF)

S1 Text Parameters used for running programs. (DOCX)
