# Supplementary material for: Phylogenic inference using alignment-free methods for applications in microbial community surveys using 16s rRNA gene
Source: PLoS One. 2017 Nov 14;12(11):e0187940. doi: 10.1371/journal.pone.0187940 (PMC5685621; doi:10.1371/journal.pone.0187940)
Supplement: S1 Fig — (PDF) [file pone.0187940.s001.pdf]

5850	5850	5850	Taxonomic
11359	10311	Kr	
11359	CVTree		
ACS			

Supplemental Figure S1. Average tree distances between alignment-free methods and the taxonomic gold-standard in the data from HMP stool samples.
