# Supplementary material for: Phylogenic inference using alignment-free methods for applications in microbial community surveys using 16s rRNA gene
Source: PLoS One. 2017 Nov 14;12(11):e0187940. doi: 10.1371/journal.pone.0187940 (PMC5685621; doi:10.1371/journal.pone.0187940)
Supplement: S1 Text — (DOCX) [file pone.0187940.s002.docx]

**S1 Text. Parameters used for running programs**

#Running NEIGHBOR

Y (accept default setting)

Default setting:

Settings for this run:

N Neighbor-joining or UPGMA tree? Neighbor-joining

O Outgroup root? No, use as outgroup species 1

L Lower-triangular data matrix? No

R Upper-triangular data matrix? No

S Subreplicates? No

J Randomize input order of species? No. Use input order

M Analyze multiple data sets? No

0 Terminal type (IBM PC, ANSI, none)? ANSI

1 Print out the data at start of run No

2 Print indications of progress of run Yes

3 Print out tree Yes

4 Write out trees onto tree file? Yes

#Running CONSENSE

1

2

3

Y (changed option 1, 2, 3 to not print out the set of species, print the indication of progress of run, or print the trees, kept everything else to default)

Settings for this run:

C Consensus type (MRe, strict, MR, Ml): Majority rule (extended)

O Outgroup root: No, use as outgroup species 1

R Trees to be treated as Rooted: No

T Terminal type (IBM PC, ANSI, none): ANSI

1 Print out the sets of species: No

2 Print indications of progress of run: No

3 Print out tree: No

4 Write out trees onto tree file: Yes

#Running TREEDIST

D (changed from branch score distance to symmetric difference)

2 (went into tree pairing submenu)

P (changed tree pairing to distance between all possible pairs in tree file)

F (full matrix for output)

Y

Settings for this run:

D Distance Type: Symmetric Difference

R Trees to be treated as Rooted: No

T Terminal type (IBM PC, ANSI, none): ANSI

1 Print indications of progress of run: Yes

2 Tree distance submenu: Distances between all possible pairs in tree file.

#Running FITCH

Y (accept default setting)

Settings for this run:

D Method (F-M, Minimum Evolution)? Fitch-Margoliash

U Search for best tree? Yes

P Power? 2.00000

- Negative branch lengths allowed? No

O Outgroup root? No, use as outgroup species 1

L Lower-triangular data matrix? No

R Upper-triangular data matrix? No

S Subreplicates? No

G Global rearrangements? No

J Randomize input order of species? No. Use input order

M Analyze multiple data sets? No

0 Terminal type (IBM PC, ANSI, none)? ANSI

1 Print out the data at start of run No

2 Print indications of progress of run Yes

3 Print out tree Yes

4 Write out trees onto tree file? Yes

RAxML was run on HPC using command line parameters: “raxmlHPC-SSE3 -m GTRGAMMA -p 12345 - -# 20.”
